# Four in ten married women demands satisfied by modern contraceptives in high fertility sub-Saharan Africa countries: a multilevel analysis of demographic and health surveys

**DOI:** 10.1186/s12889-022-14610-x

**Published:** 2022-11-24

**Authors:** Wubshet Debebe Negash, Tadele Biresaw Belachew, Desale Bihonegn Asmamaw, Desalegn Anmut Bitew

**Affiliations:** 1 Department of Health Systems and Policy, Institute of Public Health, College of Medicine and Health Sciences, University of Gondar, P.O.Box: 196, Gondar, Ethiopia; 2 Department of Reproductive Health, Institute of Public Health, College of Medicine and Health Sciences, University of Gondar, Gondar, Ethiopia

**Keywords:** Demand satisfied, Modern contraceptives, High fertility, Sub Saharan Africa

## Abstract

**Background:**

Demand satisfied with modern contraceptive can be seen on both a health and economic level. Additionally, family planning helps to regulate fertility, prevent unintended pregnancies and their consequences. Thus, the aim of this study was to identify the magnitude of demand satisfied with modern contraceptive among married/in-union women in ten high fertility sub Saharan African countries.

**Methods:**

Recent Demographic and Health Surveys that included a weighted sample of 43,745 women of reproductive age provided the data for this study. All statistical analyses were conducted once the data had been weighted, and Stata version 16.0 was used. A multilevel mixed-effect binary logistic regression model was fitted. To determine statistically significant individual and community-level factors associated with demand satisfied for modern contraceptive, odds ratios with a 95% confidence interval was generated. A *p*-value less than 0.05 was declared as statistical significance.

**Results:**

Overall, demand satisfied to use modern contraceptive in high fertility sub-Saharan Africa countries was 39.53% (95%CI: 39.06, 39.98). Women aged 25–34 (AOR: 1.34, 95%CI: 1.26, 1.42) and 35–49 (AOR: 1.28, 95%CI: 1.20, 1.38), women education: primary (AOR: 1.35, 95%CI: 1.27, 1.44) and secondary (AOR: 2.05, 95%CI: 1.90, 2.21), husband education: primary (AOR: 1.26, 95%CI: 1.18, 1.35) and secondary (AOR: 1.54, 95%CI: 1.43, 1.66), husband residence (AOR: 1.75, 95%CI: 1.60, 1.91), media exposure (AOR: 1.22, 95%CI: 1.15, 1.29), wealth index: poorer (AOR: 1.1, 95%CI: 1.02, 1.19), middle (AOR: 1.18, 95%CI: 1.08, 1.28), richer (AOR: 1.37, 95%CI: 1.26, 1.49) and richest (AOR: 1.34, 95%CI: 1.56, 1.89), number of children: 4–6 (AOR: 0.48, 95%CI: 0.43, 0.55) and above 6 (AOR: 0.39, 95%CI: 0.29, 0.59), perceived distance to the health facility not big problem (AOR: 1.11, 95%CI: 1.04, 1.15), urban residence (AOR: 1.18, 95%CI: 1.10, 1.27), high community level poverty (AOR: 0.85, 95%CI: 0.74, 0.97) were significantly associated with demand satisfied for modern contraceptives.

**Conclusion:**

Only four in ten married reproductive age women demands satisfied with modern contraceptives in high fertility Sub Saharan African countries. Modern contraceptives should therefore be more widely available, especially in rural areas and for those living away from health facilities. Also, increasing media exposure and education, providing financial support, and making contraceptive access easier for married women from poor households are important interventions that need to be put in place.

## Background

Providing voluntary family planning counseling and services during pregnancy, childbirth, and postpartum periods is a crucial means of protecting women who are postpartum and post abortion and reducing unintended and closely spaced pregnancies [1, 2]. Through using family planning, it is possible to prevent 70% of maternal and 58% of under-five deaths by extending birth interval [3]. Additionally, it helps for child growth and development [4], promotes women’s autonomy in health care utilization, education, and empowerment in workforce [5–7], and can reduce 40% of unintended pregnancies [8]. Even though family planning has the aforementioned purposes, more than 200 million women in developing countries are still seeking to use contraceptives [8]. In 2017 one out of every ten married/in union women worldwide and one in five in Africa lacked access to family planning [9].

Despite the fact that access to family planning is vital for health and development programming, in sub-Saharan Africa and South Asia, the pace of these gains has slowed over the past two decades [10, 11]. The lower demand satisfied for modern contraceptive methods had multiple negative consequences, such as unwanted pregnancies, which in turn lead to maternal mortality, prenatal depression, poor child development and restriction of women from education, and work [12–14]. The Anderson-Newman behavioral model for accessing and using health services assumes that each individual's utilization of health services depends upon their predisposing factors (such as age, education), enabling factors (such as income, availability or supply of service), and need factors [15–17]. Different factors such as lack of trust in Western medicine, low socioeconomic status, proximity of family planning clinics, and lack of knowledge about modern contraceptives have all contributed to lower utilization of modern contraceptives in Africa [18, 19].

Worldwide, 214 million women had unmet need for family planning [20]. According to the world’s 2017 family planning reports, the international modern contraceptive satisfied was 78% and in Africa it ranged from 46.5% to 56% [21, 22]. Many strategies such as the 2030 Sustainable Development Goal (SDG 3 about good health and wellbeing, SDG 5 about gender equality and women empowerment), the 2010 Every woman Every child Global initiatives, engagement of 120 million additional modern contraceptive users from 69 poorest countries by the year 2020 have been implemented to minimize the deaths of women, children, and adolescents [23–25]. Beside these important initiatives had been implemented, demand satisfied for modern contraceptive methods remained an important but continued to be less studied issue in the high fertility sub-Saharan African countries [21, 26].

Modern contraceptives have been studied from different perspectives, such as intention to use [27, 28], and utilization [6, 24, 29]. Additionally, studies were conducted within SSA on mDFPS [22, 25, 30]. However, these studies did not consider the community level variables such as community level education, community level poverty, and community level media exposure to predict mDFPS. Moreover, to the investigators knowledge, studies that combine specific high fertility countries in SSA have not been carried out to understand why married/ in union women in those specific high fertility countries have a low mDFPS. Hence, this study tried to fill this gap by using a different method of statistical analysis called multilevel mixed effect analysis, which considers both individual and community level factors.

Once the mDFPS and factors associated with it are known, interventions aimed at the family planning had showed that improvements in maternal and child health, the physical and economic wellbeing of women and their families as well as for the countries [4, 8, 22]. Assessing the actual use and associated factors of mDFPS in the high fertility sub Saharan African countries using a mixed model approach generates powerful information that triggers policy makers. Aside from improving the health of women and children, evidence-based strategies should be designed to maintain population growth. Therefore, this study tried to assess the magnitude of mDFPS and associated factors among married/in union women in high fertility sub-Saharan African countries.

## Methods

### Study design and settings

A community-based cross-sectional survey was conducted between January 2010 and December 2018 among reproductive-age women in high fertility countries in SSA. The survey was conducted in Niger, Democratic of Republic Congo, Mali, Chad, Angola, Burundi, Nigeria, Gambia, and Burkina Faso. These countries were selected because they are the top ten countries with high fertility rates in SSA, with fertility rates above 5.0, a value that is higher than the rate of 4.44 in Africa and 2.47 in worldwide [31]. Despite Somalia being one of the top ten high fertility countries, the country has no DHS data and excluded from the analysis.

The data for these countries were obtained from the official database of the DHS program, https://dhsprogram.com after authorization was granted via online request by explaining the purpose of our study. We used the woman’s individual record (IR file) data set and extracted the dependent and independent variables. DHS is a nationally representative household survey that is conducted across low and middle-income countries every five years. ICF implements the DHS Program, which aims to collect, analyze, and disseminate data about population, health, and nutrition, as well as use these data for planning, policy-making, and program management.

**Fig.1:**
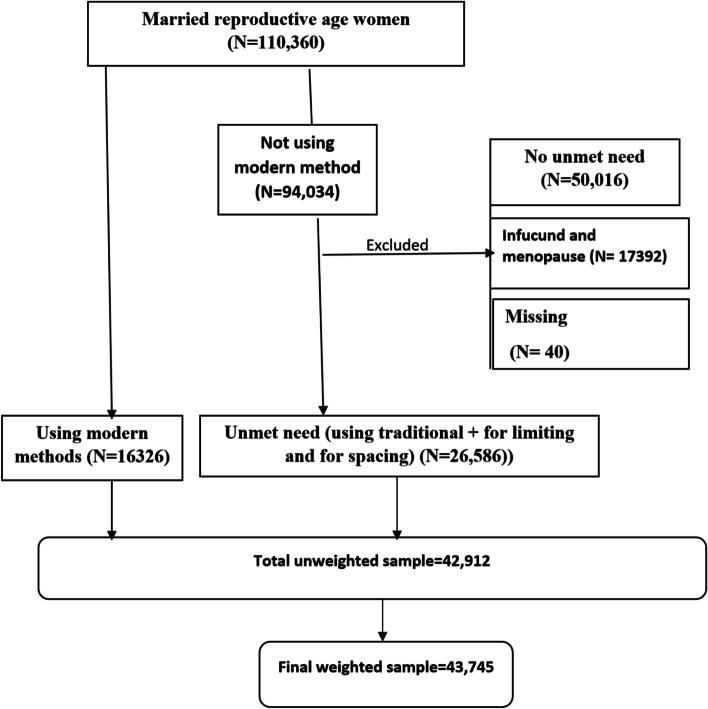
schematic presentation for demand satisfied for modern contraceptives among reproductive age women in high fertility sub-Saharan Africa countries [33]

It has been an essential data source on issues of reproductive health in low and middle income countries as it gathers data on a number of reproductive health issues [32]. A two-stage stratified sampling technique was used; a total weighted sample of 43,745 married/ in union reproductive age women were included in the study (Fig. 1).

### Study variables

Demand satisfied for modern contraceptives among married or in union reproductive age (15–49 years) women was the dependent variable. As part of this measure, women who reported using modern contraceptive methods like female sterilisation, male sterilisation, pill, intrauterine device (IUD), injectables, implants, male condom, female condom, emergency contraceptives or lactation amenorrhea methods were considered as demand satisfied by modern methods. Demand satisfied was computed using the revised definition of demand satisfied for modern contraceptives in the Demographic and Health Survey (DHS). The calculations were carried out using modern family planning methods as nominators, which are considered as met needs. Unmet needs include those who need modern contraceptive methods for spacing and/or limiting but cannot obtain them, as well as those who use traditional methods. We used the total demanded as the denominator (sum of met and unmet needs). The demand satisfied rate is equal to $\frac{met need \times 100}{unmet need+met need}$ [33]. Independent variables grouped under the individual, household and community level were included. The individual level factors were age, occupation, educational level, wealth index, media exposure, number of children, knowledge about family planning. Accordingly, age was grouped as 15–24, 25–34, and 35–49. Occupation was coded as working and not working. No formal education, primary education, secondary and higher education were the categories for highest educational level for the mother and her husband. In DHS wealth index was developed by principal component analysis using durable assets ownership, housing characteristics and access to utilities. Finally the wealth index was recoded as poorest, poorer, middle, richer, and richest. Less than five and five and above were categories for household members, number of children in the household was categorized as 0, 1–3, 4–6, and above 6, sex of household head was grouped as male or female. Knowledge about family planning was coded as ‘yes’ for those women who knows about family planning and otherwise ‘no’. Those women who were either reading newspapers/magazine, or listening radio and watching television less than once a week/at least once a week were considered as having media exposure whereas those women who had not either reading magazine/ newspaper or listening radio/ television at all was considered as having not media exposure. The community level variables were place of residence, countries, community level education, community level poverty, community level media exposure, and distance to the health facility. Thus, place of residence (urban, rural), countries, distance to the health facility (big problem, not big problem) variables were analyzed based on their categorization in the DHS [34–37]. The community level poverty, community level education and community level media exposure were generated by aggregating the individual level factors independently at cluster level and finally, were categorized as high if the proportion is ≥ 50% and low if the proportion is < 50% based on the national median value since these were not normally distributed [38].

### Modeling approaches

A multilevel logistic regression model was used to identify the association between the individual and community level factors with demand satisfied for modern contraceptive methods. STATA version 14 command “melogit” was used in fitting the models. The data was weighted (v005/1000000) throughout the analysis to ensure that the DHS sample was a representative sample and to obtain reliable estimates and standard errors before data analysis.

The first step was a graphical representation of the demand for modern contraceptives among reproductive age women. Overall, a total weighted sample of 44,052 reproductive age women were included in this study.

The second step was a bivariable analysis that calculated the proportion of demand satisfied for modern contraceptives across the independent variables with their *p*-values. All the variables having a *p*-value less than 0.2 in bivariable analysis were used for multivariable analysis. For the multivariable analysis, adjusted odds ratios with 95% confidence intervals and a *p*-value of less than 0.05 were used to identify statistically significant factors associated with demand satisfied for modern contraceptives. In the final step of the analysis, a multilevel logistic regression analysis comprising fixed effects and random effects was conducted.

The results of the fixed effects of the model were presented as adjusted odds ratio (AOR) while the random effects were assessed with intra-class correlation coefficient (ICC) [39]. Accordingly, four models were fitted; null model (model 0) which shows the variations in the demand satisfied on modern contraceptives in the absence of any independent variables. Model II adjusted for the individual-level variables, Model III adjusted for the community level variables, and model IV adjusted for both individual and community level variables [39, 40]. Correspondingly, model fitness was done using the deviance (-2 log likelihood). Variance inflation factor (VIF) was used to check for multicollinearity among independent variables in which the result showed no multicollinearity (mean value for the final model = 1.38).

## Results

### Sample characteristics/Individual and community level characteristics of reproductive age women

In this study a total weighted sample of 43,745 reproductive age women were participated. The majority (42.99%) of the study participants were in the age group of 25–34 years with a mean age of 31.14 ± 7.85 years. Of the study participants, 44.56% of the women had no formal education. Most (70.12%) of the women had work. Among the women surveyed, 87.93% of the household head were males. Of the study participants, 62.11% were from five and more household members. More than half (54.32%) of the women were from community having low proportion of education. With regard to community level poverty, most (88.16%) were from communities having low proportion of poverty (Table 1).

**Table 1:** Individual and community level characteristics of study participants in high fertility sub Saharan Africa countries (*n* = 43,745)

**Variables**	**Categories**	**Frequency (%)**	**Weighted mDFPS estimate**
Age in years	15–24	9576(21.89)	3231(33.74)
	25–34	18,806(42.99)	7792(41.43)
	35 -49	15,363(35.12)	6269(40.80)
Household wealth index	Poorest	6862(15.69)	1750(25.50)
	poorer	7853(17.95)	2311(29.43)
	Middle	8547(19.54)	2914(34.10)
	Richer	9620(21.99)	4139(43.02)
	Richest	10,863(24.63)	6177(56.86)
Educational status of the women	No formal education	19,492(44.56)	5658(29.03)
	Primary	10,936(25.00)	4255(38.91)
	Secondary and higher	13,317(30.44)	7378(55.40)
Husband education	No formal education	17,657(40.40)	5144(29.13)
	Primary	8499(19.45)	3377(39.74)
	Secondary and higher	17,551(40.16)	8757(49.89)
Occupational status of the women	Working	30,632(70.12)	12,821(41.86)
	Not working	13,055(29.88)	4456(34.14)
Number of household members	Below 5	16,574(37.89)	7268(43.85)
	5 and above	27,171(62.11)	10,023(36.89)
Number of children in the household	0	3899(8.91)	2229(57.17)
	1–3	36,000(82.29)	14,029(38.97)
	4–6	3433(7.85)	949(27.64)
	Above 6	414(0.95)	85(20.46)
Sex of household head	Male	38,464(87.93)	15,455(40.18)
	Female	5281(17.07)	1836(34.77)
Media exposure	Yes	29,241(67.01)	13,133(44.91)
	No	14,398(32.99)	4120(28.61)
Distance to the health facility	Big problem	14,363(34.76)	4869(33.90)
	Not big problem	26,951(65.24)	11,991(44.49)
Residence	Urban	16,651(38.06)	8206(49.28)
	Rural	27,094(61.94)	9086(33.53)
Community level education	High	19,982(45.68)	10,500(44.19)
	Low	23,763(54.32)	6791(33.99)
Community level poverty	High	5177(11.84)	1489(28.77)
	Low	38,568(88.16)	15,802(40.97)
Community level media exposure	High	21,775(49.78)	9628(43.82)
	Low	21,970(50.22)	7663(35.18)

**Fig. 2:**
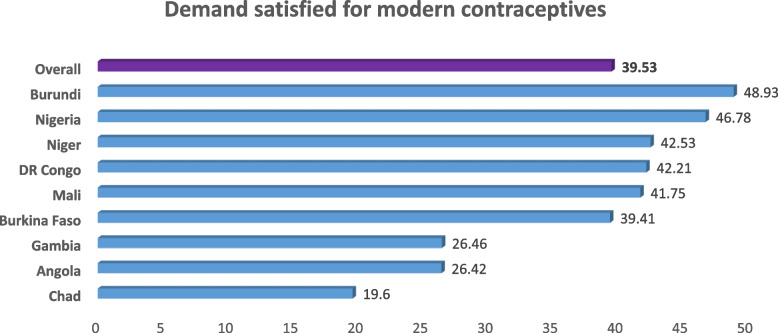
Magnitude of demand satisfied for modern contraceptives in high fertility sub-Sahara African countries

The overall magnitude of demand satisfied for modern contraceptive methods in the high fertility countries in sub Saharan Africa was 39.53% (95%CI: 39.06, 39.98). The highest (48.93%) mDFPS methods was in Burundi and Chad scoring the lowest (19.6%) prevalence (Fig. 2).

### Random effects (measure of variations) results

The null model in the random effects, revealed a statistically significant difference in the likelihood of demand satisfied for modern contraceptive methods with a community variance of 49.3%. Additionally, the intra-class correlation coefficient (ICC) in the null model showed that 13.0% of the total variability of demand satisfied for modern contraceptive accounted for differences among clusters. Further evidence of variation in the demand satisfied for modern contraceptive techniques was provided by the median odds ratio. Accordingly the odds of demand satisfied for modern contraceptives was 1.24 times higher among women of higher cluster of demand satisfied for modern contraceptives than women within lower cluster of demand satisfied for modern contraceptives. In terms of model comparison, model three was selected as a final model since it has the lowest deviance (48,857.554) (Table 2).

**Table 2:** Multilevel analysis of factors associated with demand satisfied for modern contraceptives among reproductive age women in high fertility sub Saharan Africa countries (*n* = 43,745)

Variables	Categories	Demand satisfied		Model II AOR (95% CI)	Model III AOR (95% CI)	Model IV AOR (95% CI)
		Yes n (%)	No n (%)			
Study year	2010–2013	5965(39.80)	9258(60.82)	1		1
	2014–2015	1806(24.95)	5434(75.05)	0.52(0.49, 0.56)		0.99(0.84, 1.18)
	2016–2018	9520(44.73)	11,762(55.27)	1.01(0.96, 1.06)		0.77(0.61, 1.01)
Age in years	15–24	3230(33.74)	6345(66.26)	1		**1**
	25–34	7792(41.43)	11,014(58.57)	1.35(1.27, 1.43)		1.34(1.26, 1.42)*
	35–49	6269(40.80)	9095(59.20)	1.28(1.19, 1.36)		1.28(1.20, 1.38)*
Women educational level	No formal education	5658(29.03)	13,834(70.97)	1		**1**
	Primary	4255(38.91)	6680(61.09)	1.32(1.25, 1.41)		1.35(1.27, 1.44)*
	Secondary and higher	7378(55.40)	5939(44.60)	1.83(1.70, 1.96)		2.05(1.90, 2.21)*
Husband educational level	No formal education	5144(29.13)	12,513(70.87)	1		1
	Primary	3377(39.74)	5122(60.26)	1.31(1.22, 1.39)		1.26(1.18, 1.35)*
	Secondary and higher	8757(49.89)	8794(50.11)	1.33(1.25, 1.42)		1.54(1.43, 1.66)*
Husband residence	Living elsewhere	1767(31.95)	3764(68.05)	1		1
	Living with her	15,511(40.65)	22,648(59.35)	1.66(1.53, 1.81		1.75(1.60, 1.91)*
Media exposure	No	4120(28.61)	10,278(71.39)	1		1
	Yes	13,133(44.91)	16,108(55.09)	1.15(1.09, 1.21)		1.22(1.15, 1.29)*
Wealth index	Poorest	1750(25.50)	5112(74.50)	1		1
	Poorer	2311(29.43)	5542(70.57)	1.10(1.02, 1.19)		1.10(1.02, 1.19)*
	Middle	2914(34.10)	5632(65.90)	1.23(1.13, 1.33)		1.18(1.08, 1.28)*
	Richer	4139(43.02)	5482(56.98)	1.49(1.37, 1.61)		1.37(1.26, 1.49)*
	Richest	6177(56.86)	4686(43.14)	2.10(1.93, 22.28)		1.72(1.56, 1.89)*
Household head	Male	15,455(40.18)	23,009(59.82)	1		1
	Female	1836(34.77)	3445(65.23)	0.96(0.88, 1.04)		1.02(0.93, 1.12)
Number of HH member	< 5	7268(43.85)	9306(56.15)	1		1
	≥ 5	10,023(36.89)	17,148(63.11)	0.83(0.79, 0.88)		0.85(0.81, 0.89)*
Number of children	0	2229(57.17)	1670(42.83)	1		1
	1–3	14,029(38.97)	21,971(61.03)	0.58(0.54, 0.63)		0.59(0.54, 0.63)*
	4—6	949(27.64)	2484(72.36)	0.44(0.39, 0.49)		0.48(0.43, 0.55)*
	Above 6	85(20.46)	329(79.54)	0.27(0.21, 0.36)		0.39(0.29, 0.59)*
Distance to health facility	Big problem	4869(33.90)	9494(66.10)		1	1
	Not big problem	11,991(44.49)	14,961(55.51)		1.27(1.21, 1.33)	1.11(1.04, 1.15)*
Residence	Rural	9086(33.53)	18,009(66.47)		1	1
	Urban	8206(49.28)	8445(50.72)		2.33(2.21, 2.46)	1.18(1.10, 1.27)*
Community level education	Low	6791(33.99)	13,191(66.01)		1	1
	High	10,500(44.19)	13,263(55.81)		1.26(1.15, 1.39)	1.04(0.94, 1.14)
Community level poverty	Low	15,802(40.97)	22,766(59.03)		1	1
	High	1489(28.77)	3688(71.23)		0.70(0.61, 0.81)	0.85(0.74, 0.97)*
Community level media exposure	Low	7663(35.19)	14,112(64.81)		1	1
	High	9628(43.82)	12,342(56.18)		1.24(1.14, 1.34)	1.03(0.95, 1.12)
Countries	Niger	1171(42.53)	1582(57.47)		1	1
	Angola	1085(26.42)	3023(73.58)		0.29(0.26, 0.32)	0.27(0.20, 0.32)*
	Burkina Faso	2161(39.41)	3324(60.59)		0.82(0.74, 0.91)	0.74(0.66, 0.89)*
	Burundi	2784(48.93)	2907(51.07)		1.41(1.28, 1.56)	1.30(1.00, 1.70)*
	DR. Congo	2444(42.21)	3347(57.79)		0.76(0.69, 0.84)	0.57(0.50, 0.65)*
	Gambia	609(26.46)	1692(73.54)		0.31(0.27, 0.35)	0.31(0.27, 0.36)*
	Mali	1465(41.75)	2044(58.25)		0.93(0.83, 1.04)	1.05(0.80, 1.37)
	Nigeria	4829(46.78)	5494(53.22)		0.74(0.67, 0.82)	0.63(0.48, 0.82)*
	Chad	741(19.60)	3042(80.40)		0.37(0.32, 0.43)	0.36(0.29, 0.46)*
Random effect	Null model			Model II	Model III	Model IV
Variance		0.493		0.262	0.272	0.229
ICC (%)		13.0		7.3	7.6	6.5
MOR		1.81		1.32	1.35	1.24
PCV		*Ref*		46.86	44.82	53.54
Model comparission						
Deviance(2loglikelihood)		56,861.138		51,547.446	51,798.038	48,857.554
Mean VIF		…		1.70	1.84	1.38

*ICC* Intra class corrolation cofficent, *MOR* Median odds ratio, *PCV* Proportional change in variance, *AOR* Adjusted odds ratio, *CI* Confidence interval, *VIF* Variance Inflation Factor

*P-value < 0.05

### Fixed effects (measure of associations) results

Table 2 demonstrates binary logistic regression for factors associated with mDFPS. After adjusting for individual and community related factors of demand satisfied for modern contraceptive methods, age of the women, women and husband education, husband residence, media exposure, wealth index, the number of children from the individual level factors and distance to the health care facility, urban residence, community poverty and country from the community level variables were statistically, significant associated factors with demand satisfied for modern contraceptive.

The odds of demand satisfied for modern contraceptive methods among women aged 25–34 and 35–49 years had 1.34 (AOR: 1.34, 95%CI: 1.26, 1.42) and 1.28 (AOR: 1.28, 95%CI: 1.20, 1.38) times higher as compared to women aged 15–24 years, respectively.

The likelihood of demand satisfied for modern contraceptive was 1.35 (AOR: 1.35, 95%CI: 1.27, 1.44) times and two times (AOR: 2.05, 95%CI: 1.90, 2.21) higher among women who had educated primary and secondary and higher education, respectively. Similarly, the odds of demand satisfied for modern contraceptive was 1.26 (AOR: 1.26, 95%CI: 1.18, 1.35) and 1.54 (AOR: 1.54, 95%CI: 1.43, 1.66) times higher among women whose husband had educated primary and secondary education than those women whose husband had no formal education, respectively.

The odds of demand satisfied to use modern contraceptives among women who lives with their husband was nearly two times higher as compared to their counterparts (AOR: 1.75, 95%CI: 1.60, 1.91). The likelihood of demand satisfied for modern contraceptives was 1.22 (AOR: 1.22, 95%CI: 1.15, 1.29) times higher among women who had media exposure as compared to women who had no media exposure (AOR: 1.22, 95%CI: 1.15, 1.29).

The odds of demand satisfied was 1.1 (AOR: 1.1, 95%CI: 1.02, 1.19), 1.18 (AOR: 1.18, 95%CI: 1.08, 1.28), 1.37(AOR: 1.37, 95%CI: 1.26, 1.49), and 1.72(AOR: 1.72, 95%CI: 1.56, 1.89) times higher among poorer, middle, richer, and richest households as compared to the poorest wealth quintile households.

The odds of demand satisfied for modern contraceptives among women who had 4–6 and above 6 children was reduced by 52% (AOR: 0.48, 95%CI: 0.43, 0.55) and 61% (AOR: 0.39, 95%CI: 0.29, 0.59) as compared to those women who had below 4 children, respectively.

Women who perceived distance to the health facility as not a big problem had 1.11 times higher odds (AOR: 1.11, 95%CI: 1.04, 1.15) of demand satisfied than their counterparts.

Urban resident women had 1.18 times higher odds (AOR: 1.18, 95%CI: 1.10, 1.27) of demand satisfied to use modern contraceptives than rural resident women.

Women from high community poverty had 15% less odds (AOR: 0.85, 95%CI: 0.74, 0.97) of demand satisfied for modern contraceptive methods as compared to women from low community poverty.

Demand satisfied for modern contraceptive methods was 73% (AOR: 0.27, 95%CI: 0.20, 0.32), 26% (AOR: 0.74, 95%CI: 0.66, 0.89), 43% (AOR: 0.57, 95%CI: 0.50, 0.65), 69% (AOR: 0.31, 95%CI: 0.27, 0.36), 37%(AOR: 0.63, 95%CI: 0.48, 0.82), 52% (AOR: 0.48, 95%CI: 0.43, 0.55) less odds and 1.3 times higher odds (AOR: 1.30, 95%CI: 1.00, 1.70) in Angola, Burkina Faso, Democratic Republic Congo, Gambia, Nigeria, Chad and Burundi as compared to Niger, respectively (Table 2).

## Discussion

The study identified demand satisfied for modern contraceptive and associated factors among married women in high fertility sub Saharan Africa. The finding of this study reveals that almost, four among ten, 39.53% (95%CI: 39.06, 39.98) women had demand satisfied for modern contraceptive.

Demand satisfied for modern contraceptive methods was 73%, 26%, 43%, 69%, 37%, 64% less odds, and 30% higher odds in Angola, Burkina Faso, Democratic Republic Congo, Gambia, Nigeria, Chad and Burundi as compared to Niger, respectively. The better demand satisfaction for modern contraceptive in Niger might possibly the government’s ongoing improvement, such as a segmentation counseling strategy that has been developed by the Niger government to increase the use of family planning; this segmentation approach identifies categories of women and makes counseling specific to their needs. This approach will support the goal that all women being introduced to a range of contraceptive methods to meet their current family planning needs [41, 42]. Additionally, community based family planning interventions in Niger are a very important strategy to improve met need [43].

The overall magnitude of demand satisfied for modern contraceptive is in line with a study conducted in Ethiopia 39.5% [21]. However, our finding is lower than studies conducted in low and middle income countries 52.9% [4] and among 185 countries 75.8% [24] and the focus countries of 2020 family planning (67.9%) [25], and in Jordan 54.7% [26]. The lower demand satisfied in our study might be accounted with the continuous increase in the absolute number of women of reproductive age in sub-Saharan Africa. Here, the percentage of women with unmet need for family planning is increased as well. Adolescents in Sub-Saharan Africa, in particular, have a significant unmet need for sexual and reproductive health care, whereas in most other regions of the world, the number of adolescent girls and young women with an unmet need for contraception has decreased or remained constant [44, 45]. Additionally, in Africa one among five women lack access to family planning since 2017 [9].This study tried to assess demand satisfied for modern contraceptives among married women in selected high fertility countries. Which indicates a need to overcome unmet need and extensive women’s counseling to bring down population growth and unwanted or unplanned pregnancies.

The result of this study revealed that higher odds of demand satisfied for modern contraceptive methods in the oldest age groups of women as compared to those young women. On the contrary higher odds of mDFPS was observed among young women in Ethiopia [46], Uganda [47], Zambia [29], Bangladesh [48]. The reason might be accounted that limited knowledge, access, worries about fertility and low status of women are the major factors of family planning utilization among youths in developing country [49]. On the other hand those older women might receive enough information about family planning from relatives, and media in their lives [50]. Moreover, these older women had higher desire in spacing births. On the contrary those young women are a high desire in bearing children [51]. This implies that improving modern contraceptive access and counseling of young women might be very important to address unmet need.

The odds of demand satisfied for modern contraceptive was higher among women who had educated as compared to women who had no formal education. Similarly, women whose husband had educated primary and secondary education were higher odds of demand satisfied for modern contraceptives than women whose husband had no formal education. Perhaps this is due to the fact that education enhances the self-confidence of women, gives them a better understanding of health care decision-making, and helps them make independent decisions about their health care utilization. As a result of exercising gender equality, educated women are more likely to participate in health care decisions [52, 53]. Additionally, if partner is educated, the more he will accept gender equality and believe in equal participation in decision making [54, 55].

The odds of demand satisfied to use modern contraceptives was nearly twice among women who lives with their husband as compared to those women who lives separately with their husband. The same is true in Nepal [56, 57]. Women who live with their husbands probably have frequent sexual contact, so they may believe pregnancy is likely, leading them to look for family planning. Therefore, family planning can be of greater benefit to them, such as minimizing unwanted and unplanned pregnancies.

Women who had media exposure had higher odds of demand satisfied for modern contraceptives as compared to women who had no media exposure. This finding is consistent with studies done in Ethiopia [58], Nigeria [59], and Pakistan [60]. The possible reason for this finding is women with high media exposure might have a better understanding of reproductive health rights and the advantages of their health care service utilization that encourages their participation in reproductive health decisions [58].

The odds of demand satisfied was higher among poorer, middle, richer, and richest households as compared to the poorest wealth quintile households. The finding is similar with studies conducted in Africa [61], Nigeria [62], and Zambia [29]. The possible justification might be having enough income enables women to involve in labor force in which their level of awareness and decision making power will be improved [61]. Similarly, women from high community poverty had 15% less odds of demand satisfied for modern contraceptive methods as compared to women from low community poverty. This might be that women from the high community poverty have decreased the use of contraceptives [63]. Because the children are considered as an economic value who pay back during the old age [64]. Women from the poor community may expect that when a child grows older, he/she will be a responsibility to support the parents for his/her upbringing. Additionally, couples (or parents) who place a high value on having children will seek out larger families who will later be able to take on the role of caregiving for their parents when they are older [64].

Reproductive age women who had 4–6 and above 6 children were 52% and 61% less odds of demand satisfied for modern contraceptives as compared to those women who had below 4 children, respectively. The finding is in line with a study conducted in Malawi [51]. This is because the demand for contraceptives increases with parities after the desire family size has achieved.

Urban resident women had 1.18 times higher odds of demand satisfied to use modern contraceptives than rural resident women. This finding is similar with Zambia [29], Pakistan [65], and Bangladesh [50]. The possible reason might be that in Africa most of the population are settled in rural area where education, information about the available contraceptive method, access to family planning are obstacles [51]. Again it is confirmed from this study finding that Women who perceived distance to the health facility as not a big problem had higher odds of demand satisfied than their counterparts.

Due to the high fertility rate, sub-Saharan Africa has contributed most of the world’s unexpected population dynamics. Modern contraception plays a crucial role in helping to regulate population growth, and to improve the physical and economic wellbeing of women and their families as well as for the countries. However, in Sub-Saharan African countries with high fertility, only four out of ten married/in union women demand satisfied by modern contraceptives. Thus, thousands of reproductive age women had unmet need for modern contraceptives. In turn, this can lead to an increase in unwanted or mistimed pregnancies, and sexually transmitted infections such as HIV/AIDS. In order to combat the problem, the respective country governments, nongovernmental organizations and policy makers should try to improve access to modern contraceptive or met need for family planning more widely in the region.

### Strengths and limitations

The utilization of nationally representative data, large sample size, and analysis of the individual and community level factors were the key strengths. In order to find a more accurate result, multilevel-modeling technique accounting the survey data hierarchical nature was employed. The current study is not generalisable to all women in SSA. Moreover, the study was unable to explore cultural norms and values. The study also have limitations, because the DHS data are cross-sectional no temporal link between the independent variables and the dependent variable is demonstrated.

## Conclusion

Only four in ten married reproductive age women demands satisfied with modern contraceptives in high fertility Sub Saharan African countries. Individual level factors such as age, education, husband residence, media exposure, wealth index, number of children, and distance to the health facility, and community level factors of urban residence and community level poverty were significantly associated with demand satisfied for modern contraceptives. It is therefore, important to improve access to modern contraceptives, particularly for those living in rural areas and far from health facilities. Increasing media exposure and educate women about modern contraceptives, increasing financial support, and enabling married women from poor households to get contraceptives, are also important interventions that need to be put in place.

## Data Availability

The study used publicly available data, which can be found at the following link: https://dhsprogram.com/data/dataset_admin/login_main.cfm?CFID=39421058&CFTOKEN=a8b8a36f1fb27230-E89DAEA4-D47B-719A 9AD1F13D7D93EF8A.

## Abbreviations

AOR: Adjusted Odds Ratio
COR: Crude Odds Ratio
CI: Confidence Interval
DHS: Demographic and Health Survey
mDFPS: Demand Satisfied for Modern Contraceptives
PCV: Proportional change in variance
ICC: Intra class corrolation cofficent
IUD: Intrauterine Device
MOR: Median Odds Ratio
SSA: Sub Saharan Africa
VIF: Variance Inflation Factor
